# Unraveling the molecular mechanisms of DNA capture by the Com pilus in naturally transformable monoderm bacteria

**DOI:** 10.1128/mbio.00851-25

**Published:** 2025-05-23

**Authors:** Jérémy Mom, Odile Valette, Laetitia Pieulle, Vladimir Pelicic

**Affiliations:** 1 Laboratoire de Chimie Bactérienne, Aix-Marseille Université/CNRS (UMR7283), Institut de Microbiologie de la Méditerranée 56185, Marseille, France; GSK Vaccines, Siena, Italy

**Keywords:** natural transformation systems, genetic competence, type 4 pili, DNA-binding proteins, gram-positive bacteria

## Abstract

Transformation is a mechanism of horizontal gene transfer widespread in bacteria. The first step in transformation—capture of exogenous DNA—is mediated by surface-exposed filaments belonging to the type 4 filament (T4F) superfamily. How these protein polymers, composed of major and minor pilin subunits, interact with DNA remains poorly understood. Here, we address this question for the Com pilus, a widespread T4F mediating DNA capture in competent monoderm species. Our functional analysis, performed in *Streptococcus sanguinis*, was guided by a complete structural model of the Com pilus. We show that the major pilin ComGC does not bind DNA. In contrast, a systematic mutational analysis of electropositive residues exposed at the filament surface in the four minor pilins (ComGD, ComGE, ComGF, and ComGG) reveals that the interface between ComGD and ComGF is important for DNA capture. Sequential mutations in these two interacting subunits lead to complete abolition of transformation, without affecting piliation. We further demonstrate the physical interaction between ComGD and ComGF using disulfide crosslinking, upon mutagenesis of two strategically positioned residues into cysteines. A structural model of the Com pilus tip interacting with DNA recapitulates all these findings and highlights a novel mode of DNA-binding, conserved in hundreds of monoderm species.

**IMPORTANCE:**

Bacteria are capable of evolving and diversifying very rapidly by acquiring new genetic material via horizontal gene transfer (HGT). Transformation is a widespread mechanism of HGT, which results from the capture of extracellular DNA by surface-exposed pili belonging to the type 4 filament (T4F) superfamily. How T4F—composed of major and minor pilins—interact with DNA remains poorly understood, especially in monoderm species that use a unique T4F for DNA capture, known as Com pilus or T4dP. The significance of this work is in characterizing a novel mode of DNA-binding by showing that the interface between two minor pilins, part of a tip-located complex of four pilins—found in different T4F—has been functionalized in monoderms to capture DNA. This is an evolutionary mechanism promoting the exceptional functional versatility of T4F.

## INTRODUCTION

Bacteria are capable of evolving and diversifying very rapidly, which has allowed them to colonize and thrive even in the most extreme environments. Since they often replicate clonally by binary fission, bacterial extraordinary evolutionary potential results from their ability to acquire new genetic material by horizontal gene transfer (HGT) (1). Deciphering the mechanisms of HGT—occurring by transformation, transduction, or conjugation—has been a hot research topic for decades, also because HGT has dire consequences for human health by leading to the spread of antibiotic resistance and the evolution of more virulent strains (1).

Transformation—documented in a wide variety of bacterial species (2)—is the only mode of HGT depending exclusively on the recipient cell, which must be in a genetically programmed state called competence (3). Transformation is defined as a genetic alteration of the recipient resulting from (i) the capture of free DNA from the extracellular milieu (also known as DNA uptake), (ii) its translocation across the cytoplasmic membrane (CM), and (iii) its stable integration into the genome (3). At a molecular level, DNA capture results from extracellular DNA being bound by filaments on the cell surface (4), translocated across the outer membrane and/or the cell wall following filament retraction (4), and compacted close to the CM upon interaction with the DNA receptor ComEA (5, 6). Then, a single strand of DNA is translocated across the CM through the ComEC channel (7), while the second strand is degraded. Finally, internalized DNA is integrated into the genome by RecA-mediated strand exchange.

With only one known exception, binding of extracellular DNA—the earliest step in transformation—requires one of the several subtypes of type 4 pili (T4P) (3). T4P belong to the T4F superfamily, functionally versatile nanomachines ubiquitous in Bacteria and Archaea (8, 9) that use a conserved multi-protein machinery to assemble and operate filamentous polymers composed of type 4 pilins. Pilins are defined as major or minor according to their respective abundance in filaments (10). Although we still lack a detailed mechanistic understanding of DNA capture by T4P, the following scenario is widely admitted. As visualized in competent diderm species—that all use type 4a pili (T4aP, where “a” denotes the subtype) for DNA capture—filaments bind DNA directly (4, 11). A few pilins acting as DNA receptors have been identified, the best characterized of which are *Neisseria meningitidis* ComP (11) and *Legionella pneumophila* FimT (12). These unrelated pilins bind DNA via patches of electropositive residues exposed on the filaments' surface. ComP binds DNA via an electropositive stripe delimited by two disulfide bonds (13) and shows preference for a short sequence motif repeated hundreds of times in *Neisseria* genomes, explaining these species' uncommon preferential uptake of their own DNA (14). FimT binds DNA via a patch of conserved electropositive residues, located in a flexible/unstructured C-terminal tail (12). Subsequently, bound DNA is brought to the CM by T4aP retraction powered by the retraction motor PilT (15). Preventing pilus retraction, either by *pilT* mutagenesis (16) or by steric obstruction (4), prevents DNA uptake.

The above DNA-binding pilins are found only in a small subset of species, which suggests that other modes of DNA capture exist. In particular, no ComP or FimT homologs are present in T4dP—also known as Com pili (17)—that are found in hundreds of monoderm species (9). T4dP, which are long (18) and dynamic filaments (19), represent a minimalistic T4F (17) requiring only a set of four proteins universally conserved in this superfamily (20) for filament assembly and functioning (17). Here, using *Streptococcus sanguinis* as a model, we determined how T4dP bind DNA. We performed a functional analysis guided by a complete structural model of the filament composed of five pilins, which uncovered a novel mode of DNA-binding involving two adjacent tip-located minor pilins. We show that the resulting model is applicable to hundreds of monoderm species where T4dP are found.

## RESULTS

### T4dP display a canonical T4P structure with a filament capped by a complex of four minor pilins

Recently, using *S. sanguinis* as a model, we showed that eight *com* genes organized in two transcription units (*comGA-comGB-comGC-comGD-comGE-comGF-comGG* and *comC*) (Fig. S1A)—conserved in hundreds of species of Firmicutes (9)—are necessary and sufficient for the assembly and functioning of T4dP (17). The identification of signature domains in the corresponding proteins using InterProScan (21) and the prediction of their 3D structures using AlphaFold 2 (22) showed that they correspond to the four proteins universally conserved in T4F (17): prepilin peptidase (PPase) (ComC), extension ATPase (ComGA), platform protein (ComGB), and type 4 pilins (ComGC, ComGD, ComGE, ComGF, and ComGG). We confirmed that the last five proteins—displaying N-terminal class 3 signal peptide (SP3) found in all type 4 pilins (10) (Fig. S1B) – are processed by ComC and assembled into filaments (17). ComGC is the major pilin, while the remaining four are minor pilins much less abundant in pilus preparations. We showed that the four minor pilins interact to form a complex (17), predicted to be located at the tip of T4dP based on similarities to complexes of four minor pilins found at the tip of other better characterized T4F (23, 24).

**Fig 1:**
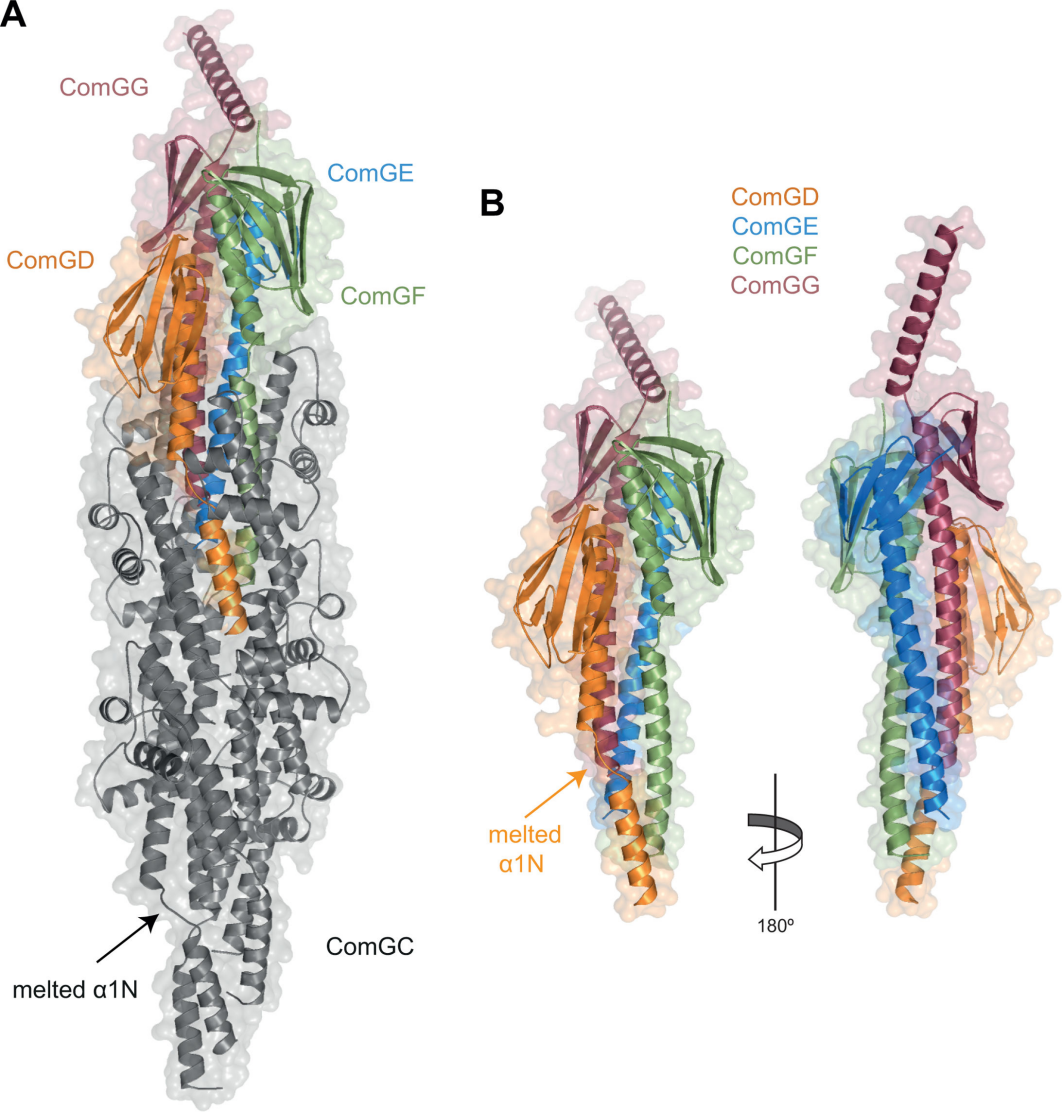
Modeling of T4dP reveals a canonical T4P, with a ComGC filament capped by a complex of four minor pilins. (**A**) Structural T4dP model in *S. sanguinis* predicted by AlphaFold 3 (25) with 10 copies of ComGC and one copy of each of the four minor pilins. The cartoon representation with surfaces shown in transparency reveals that T4dP display a canonical T4P structure, where a filament of ComGC (gray) is capped by a complex of four minor pilins ComGD (orange), ComGE (blue), ComGE (green), and ComGG (maroon). The same color code is used throughout the manuscript. As in available near-atomic resolution structures of T4aP (26–28), helical packing of ComGC subunits via their α1 helices is accompanied by “melting” of a portion of α1N that becomes non-helical. (**B**) Focus on the tip-located complex of ComGD-ComGF-ComGE-ComGG minor pilins. Two different sides are shown (180° views). As for ComGC, packing of ComGD in the pilus is accompanied by partial melting of its α1N. Intriguingly, the local confidence for the last 30 residues of ComGG, which was very low when this pilin was modeled on its own (see Fig. S2), remained very low, and the corresponding α-helix still protrudes from the tip of the pilus.

As a first step to understanding how T4dP bind DNA, we modeled the filament by taking advantage of the recent introduction of AlphaFold 3, a deep-learning framework with substantially improved prediction accuracy for complex structures (25). Using the sequence of the mature pilins of *S. sanguinis*, in which the leader peptides cleaved by ComC (Fig. S1B) were manually removed, we produced a complete structural model of T4dP. As described previously, when modeled on their own, the five pilin display typical “lollipop” architectures (10), starting with an α-helix (α1) of approximately 50 residues onto which a globular head is impaled (Fig. S2). The hydrophobic N-terminal half of α1 (α1N) protrudes from the pilin subunits. The five models exhibit excellent confidence metrics (Fig. S2), except for the last 30 residues of ComGG, for which the local confidence is very low. This portion of ComGG, modeled as an α-helix, forms an unusual protrusion from the globular head (Fig. S2). Next, we produced a complete model of a filament composed of 10 ComGC subunits and one subunit each of the four minor pilins (Fig. 1), revealing that T4dP display a canonical T4P structure (Fig. 1A). The ComGC pilus shaft is similar to near-atomic resolution T4aP structures (26–28). The major pilins pack helically with their α1 helices forming a hydrophobic core, which is accompanied by the “melting” of an α1N portion that becomes non-helical (Fig. 1A). Strikingly, the filament is capped by a ComGD-ComGF-ComGE-ComGG complex of four minor pilins (Fig. 1B), which were previously shown to interact (17). Interestingly, the packing of ComGD is accompanied by partial melting of α1N, like for ComGC (Fig. 1B). Considering its size, the confidence metrics of the T4dP model are good. The quality of the model, estimated using the Structure Assessment service (29), returned MolProbity and global QMEANDisCo scores of 2.43 and 0.67 ± 0.05, respectively. These values are comparable to those calculated for the cryo-EM structure of *S. sanguinis* T4aP (28) (MolProbity 1.12, global QMEANDisCo 0.65 ± 0.05). The local confidence for the last 30 residues of ComGG remained very low, and the corresponding α-helix protrudes from the pilus tip (Fig. 1).

Our modeling results strengthen the notion that T4dP are canonical T4P where a ComGC pilus shaft is capped by a complex of four minor pilins. This architecture is similar to that of better-characterized T4F.

### ComGC has no intrinsic DNA-binding propensity

**Fig 2:**
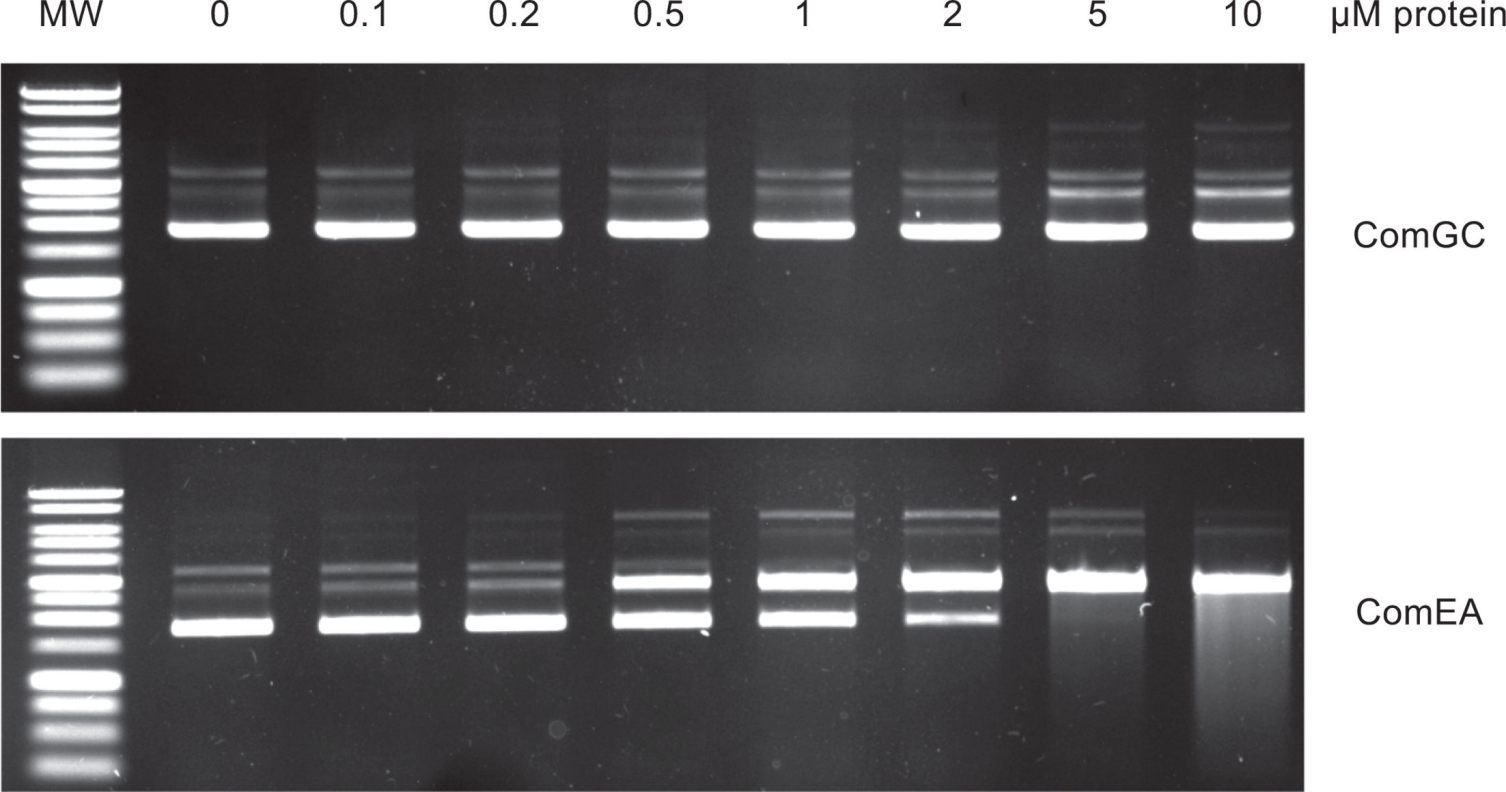
ComGC has no intrinsic DNA-binding activity. The DNA-binding propensity of purified MBP-ComGC was assessed by agarose EMSA. A standard amount (120 ng) of pUC19 plasmid was incubated with increasing concentrations of purified MBP-ComGC and resolved by electrophoresis on a 0.8% agarose gel (upper panel). As a positive control, we used purified MBP-ComEA (lower panel). ComEA is a conserved DNA receptor involved in the late stages of DNA uptake (5).

In order to understand which T4dP subunit binds DNA, we first focused on ComGC. To determine whether it has intrinsic DNA-binding activity, we tested ComGC propensity to interact with DNA using agarose electrophoretic mobility shift assays (EMSAs), as previously done for ComP from *Neisseria* (11). We used the soluble portion of ComGC—the globular head without the hydrophobic α1N, which has been characterized structurally (30)—purified as a fusion to maltose-binding protein (MBP). As target DNA, we used pUC19 plasmid. As a positive control, we used an MBP fusion to *S. sanguinis* ComEA, the DNA receptor involved in late stages of DNA uptake (5, 6). Since ComEA is a bimodular protein in *S. sanguinis* as revealed by InterProScan (21) (Fig. S3A), we fused only its C-terminal module to MBP, which is structurally similar to the crystal structure of *Thermus thermophilus* ComEA (Fig. S3B). EMSA showed that ComGC is incapable of binding DNA since no shift was seen with as much as 10 µM of purified protein (Fig. 2). In contrast, under the same conditions, ComEA bound DNA efficiently (Fig. 2). We observed a shift with as little as 0.5 µM MBP-ComEA, indicating a significant affinity for DNA, as shown in other species (5, 6). These experiments rule out the possibility that ComGC might be the primary DNA receptor during DNA capture by T4dP. This suggests that the four minor pilins capping the pilus are likely to be involved in DNA-binding.

### Surface-exposed electropositive residues in ComGD and ComGF are key for DNA capture

We next focused on the four minor pilins ComGD, ComGE, ComGF, and ComGG to understand how T4dP bind DNA. Since these proteins were recalcitrant to purification despite multiple attempts, we could not determine by EMSA whether they bind DNA. Instead, guided by our structural model, we made a series of mutations in each of the corresponding genes in *S. sanguinis* and tested their effects on transformation as a proxy for DNA binding. When phenotypic defects in transformation were observed, we ruled out that these resulted from impaired pilus biogenesis by testing the mutants for piliation. We used a previously designed pilus purification procedure (17), and detected the major pilin ComGC by immunoblotting using a specific antibody.

**Fig 3:**
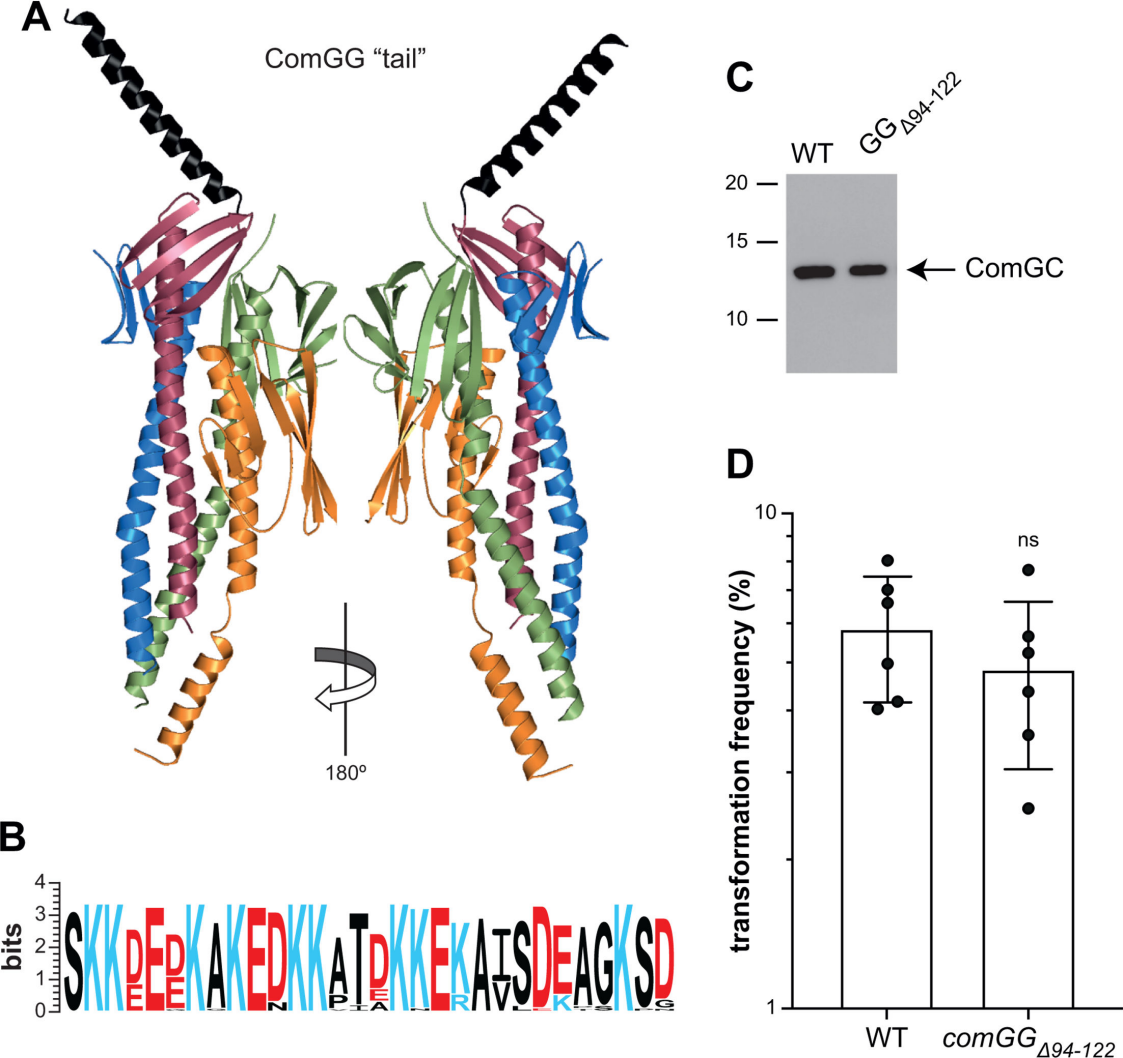
The charged C-terminal tail in ComGG is dispensable for *S. sanguinis* piliation and transformation. (**A**) Tip-located complex of four minor pilins with the last 30 residues of ComGG, which form an α-helical tail with very low local modeling confidence, highlighted in black. Two different sides are shown (180° views). (**B**) The ComGG tail is highly charged, as could be seen from the sequence logo generated from MSA of 31 *S*. *sanguinis* IPR047665 entries (*Streptococcus*-type ComGG) in InterPro (31). Charged residues are colored in blue (electropositive) or red (electronegative). (**C**) An unmarked tail-less *S. sanguinis* mutant expressing ComGG_Δ94–122_ is piliated. Piliation was assessed by immunoblotting using an anti-ComGC antibody on pilus preparations made from equal volumes of culture. The WT strain is included as a control. (**D**) The *S. sanguinis comGG_Δ94–122_* mutant is transformable. The WT strain is included as a control. Transformation frequencies (%)—mean ± SD from six independent experiments—are the ratio of transformants relative to number of viable bacteria. The *comGG_Δ94–122_* mutant is as transformable as the WT strain as assessed by a two-tailed *t*-test. ns, not statistically different.

Since FimT binds DNA via an electropositive C-terminal tail (12), we first focused on the C-terminal tail in ComGG (Fig. 3A). As confirmed by modeling other ComGG (Fig. S4A), this structural feature is widely conserved in *Streptococcus*-type ComGG (IPR047665) that are found mainly in *Streptococcaceae* and *Enterococcaceae*. As seen by aligning the sequences of ComGG tails in *S. sanguinis* proteins available in InterPro (Fig. 3B), 20 out of 30 residues are charged, 10 of which are positively charged Lys. This sequence feature is conserved in other ComGG, such as in *S. pneumoniae* proteins (Fig. S4B). We therefore engineered a mutant in *S. sanguinis* expressing a shorter ComGG, ComGG_Δ94–122_**,** in which the tail residues were truncated (the numbering is according to the processed protein). Since pilus production is abolished in a *ΔcomGG* mutant (17), we first determined whether the *comGG_Δ94–122_* mutant is piliated. Critically, we found that this mutant is unaffected for piliation despite the deletion of almost a quarter of the ComGG protein (Fig. 3C). Next, we tested whether the *comGG_Δ94–122_* mutant was still transformable by quantifying its competence. We found that this mutant was as transformable as the wild-type (WT) strain (Fig. 3D), that is, 4.84 ± 1.79% of transformed cells compared to 5.69 ± 1.43%, respectively. In contrast, we previously showed that transformation was abolished in non-piliated mutants, with decreases of at least six orders of magnitude (17). Taken together, these findings demonstrate that the charged C-terminal tail in ComGG is dispensable for DNA binding by T4dP.

**Fig 4:**
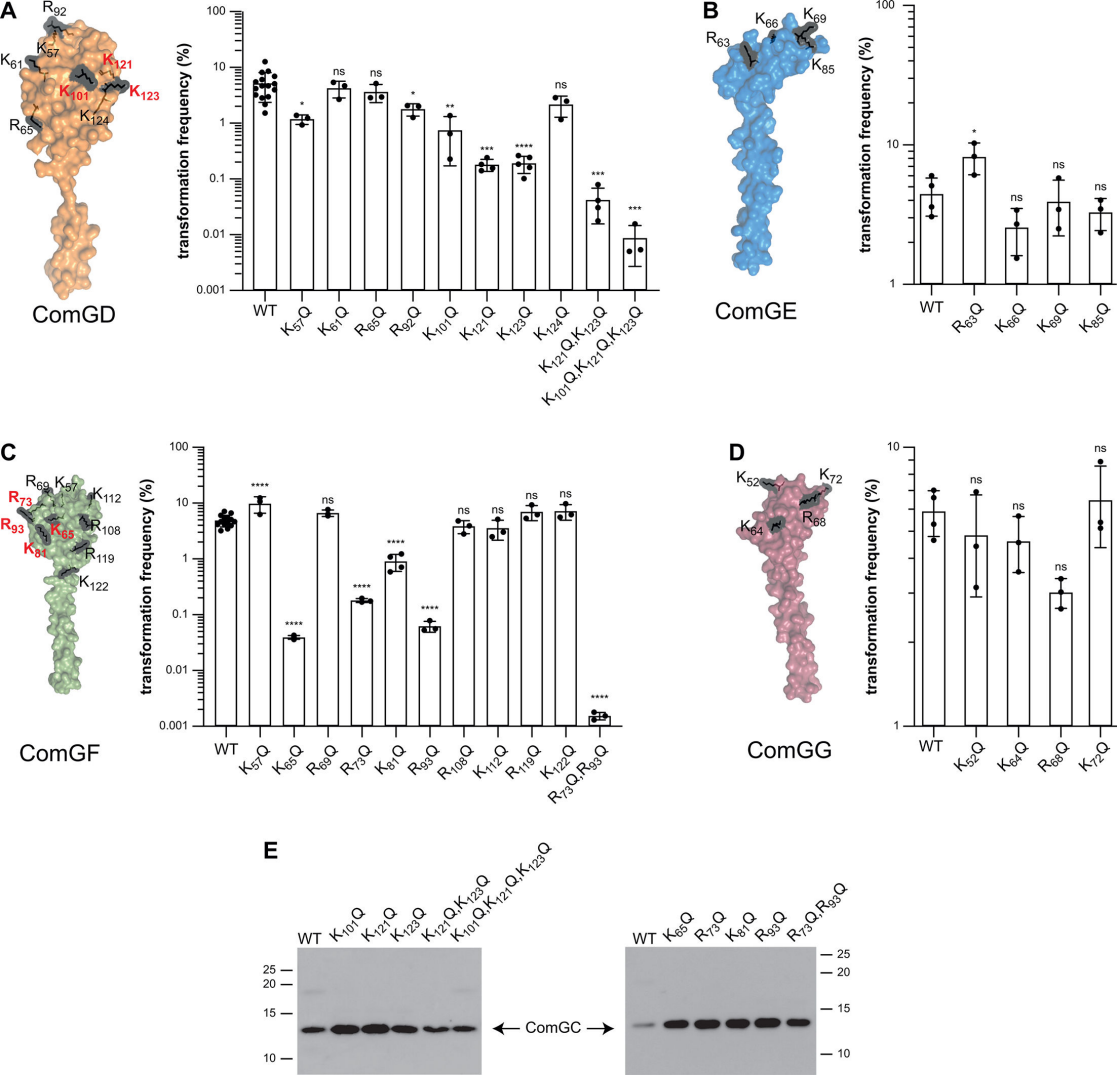
Electropositive residues in ComGD and ComGF exposed on the filament surface are key for transformation. (**A–D**) Quantifying transformation in *S. sanguinis* mutants expressing minor pilins in which we altered surface-exposed electropositive residues potentially contributing to DNA binding. The targeted residues are highlighted in black on the corresponding structures in surface representation (the C-terminal tail in ComGG is not shown). Transformation frequencies (%) were the mean ± SD from at least three independent experiments. We used Dunnett’s one-way ANOVA to compare the means to the WT. ns, not statistically different; *, *P* < 0.0332; **, *P* < 0.0021; ***, *P* < 0.0002; ****, *P* < 0.0001. (**E**) Assessing piliation in the mutants affected for transformation. This was done by immunoblotting using an anti-ComGC antibody on pilus preparations made from equal volumes of culture. The WT strain is included as a control.

Next, because the *Neisseria*-specific minor pilin ComP was reported to bind DNA via a surface-exposed stripe of electropositive residues (11), we focused on the electropositive residues in the four minor pilins predicted to be exposed on the surface of T4dP. We systematically constructed a series of *S. sanguinis* mutants in which positively charged and surface-exposed Lys and Arg residues were substituted with Gln. We started with eight mutations in ComGD and found that three mutants—K_101_Q, K_121_Q, and K_123_Q—are significantly less competent, with 10- to 100-fold decreases in transformation (Fig. 4A). Strikingly, these residues are spatially close, suggesting that they form a DNA-binding site, which was supported by combining the mutations. Indeed, ComGD polymutants—K_121_Q/K_123_Q and K_101_Q/K_121_Q/K_123_Q—displayed more drastic defects in transformation (Fig. 4A), up to a 1,000-fold. Importantly, all these mutants are piliated (Fig. 4E). For ComGE, the four mutants that we constructed were as transformable as the WT strain, suggesting that this minor pilin has no role in DNA binding (Fig. 4B). For ComGF, 4 of the 10 mutants we constructed—K_65_Q, R_73_Q, K_81_Q, and R_93_Q—were significantly less competent than WT (Fig. 4C), with up to a 1,000-fold decrease in transformation. These residues are spatially close, suggesting that they form a DNA-binding site. This was strengthened by showing that the R_73_Q/R_93_Q polymutant displayed a more drastic 10^4^-fold decrease in transformation (Fig. 4C). Importantly, all these mutants are piliated (Fig. 4E). Finally, for ComGG, none of the four mutants that we constructed—we did not target the electropositive tail residues analyzed above (see Fig. 3)—were affected for competence (Fig. 4D), suggesting that this minor pilin has no role in DNA binding.

Taken together, these findings demonstrate that electropositive residues in the ComGD and ComGF subunits of the complex of four minor pilins capping T4dP are key for competence, most likely by playing a role in binding DNA.

### The interface between ComGD and ComGF is key for DNA capture

**Fig 5:**
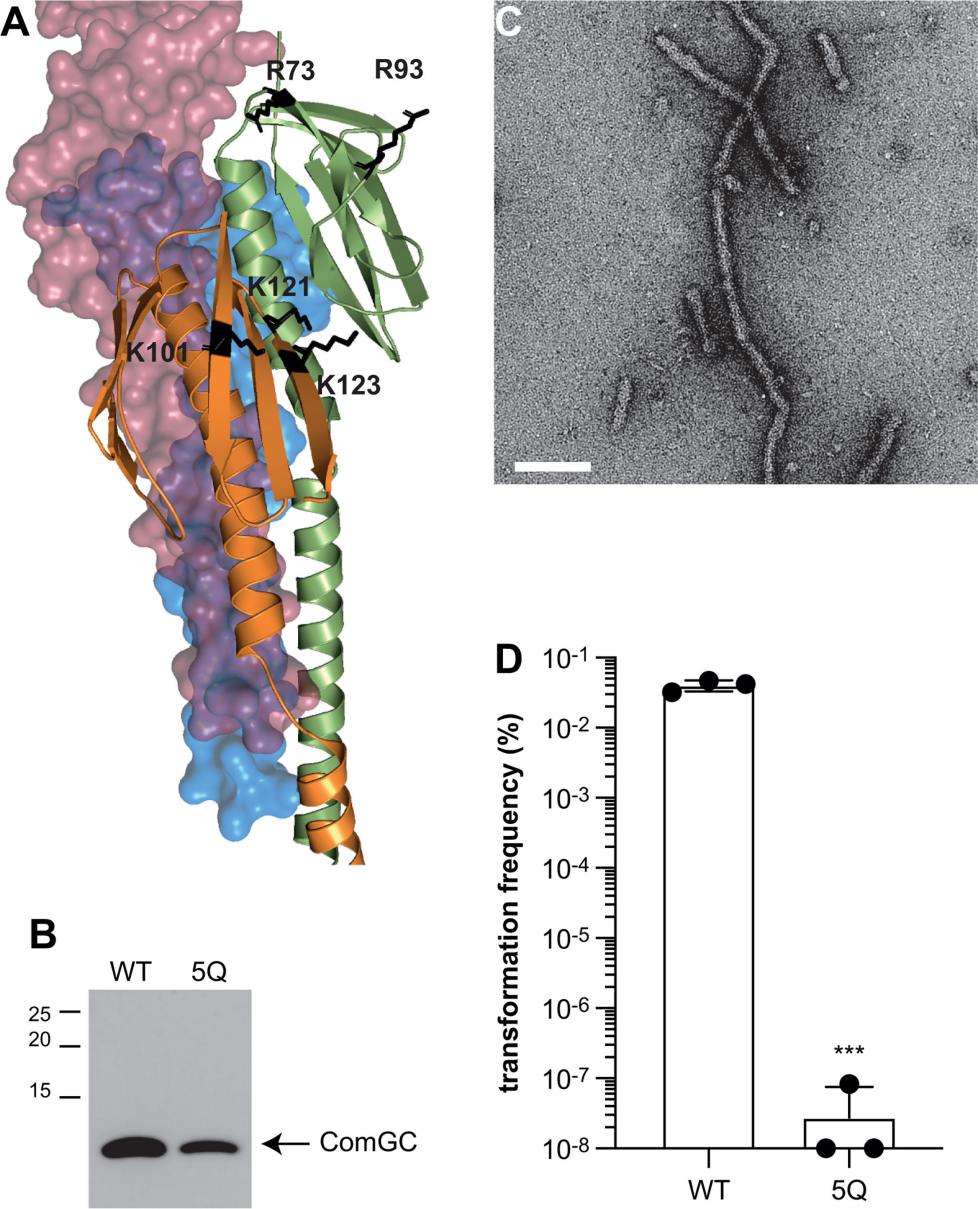
The interface between ComGD and ComGF is key for DNA capture by T4dP. (**A**) ComGD-ComGF interface at the tip of the pilus. The electropositive residues shown to be important for transformation in Fig. 4 are highlighted in black. We constructed a quintuple *S. sanguinis* ComGD_K101Q/K121Q/K123Q_ ComGF_R73Q/R93Q_ mutant—named 5Q—in which all these electropositive residues were altered simultaneously. (**B**) The 5Q mutant is piliated as assessed by immunoblotting using an anti-ComGC antibody on pilus preparations made from equal volumes of culture. The WT strain is included as a control. (**C**) Pili in the 5Q mutant are morphologically normal as assessed by TEM on purified filaments. The scale bar represents 100 nm. (**D**) Transformation is abolished in the 5Q mutant. Transformation frequencies (%) are the mean ± SD from three independent experiments. Statistical significance assessed by a two-tailed *t*-test. *** 0.0002 < *P* < 0.0021.

In the model of the pilus, ComGD and ComGF are adjacent subunits. Strikingly, the electropositive residues in these two minor pilins key for DNA capture are spatially close on one side of the pilus tip (Fig. 5A) and could together represent an electropositive DNA-binding patch. To provide evidence for this, we combined mutations in ComGD and ComGF to create the quintuple mutant ComGD_K101Q/K121Q/K123Q_ ComGF_R73Q/R93Q_, which we will refer to as 5Q for the sake of readability. We first showed by immunoblotting on pilus preparations that the 5Q mutant is piliated (Fig. 5B). Moreover, as shown by transmission electron microscopy (TEM), purified filaments displayed a morphology indistinguishable from WT filaments (17) (Fig. 5C). Critically, although the 5Q mutant pili appear normal, its transformation is abolished with more than a 10^6^-fold decrease compared to WT (Fig. 5D). Such a dramatic defect in transformation, which has previously been observed only in non-piliated mutants (17), is stronger than the decreases observed in either of the polymutants in ComGD or ComGF (see Fig. 4). Taken together, these results show that electropositive residues in ComGD and ComGF on one side of the T4dP tip constitute together an interface key for DNA capture.

**Fig 6:**
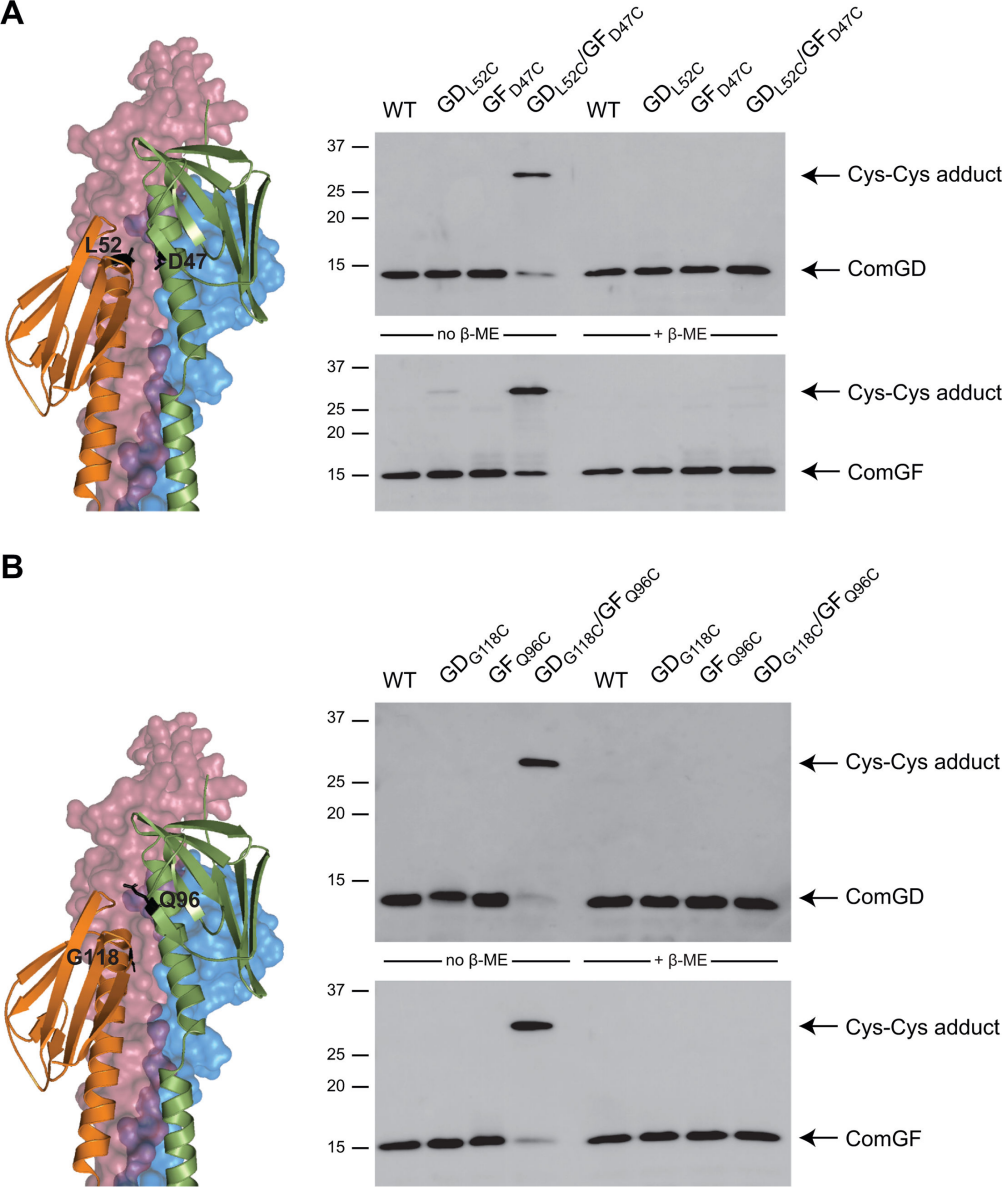
Probing the interface between ComGD and ComGF by Cys crosslinking. We constructed *S. sanguinis* single and double mutants in a strain that constitutively expresses T4dP (17), with Cys substitutions in two different pairs of residues. (**A**) ComGD_L52C_/ComGF_D47C_ and (**B**) ComGD_G118C_/ComGF_Q96C_. Disulfide crosslinking was tested in the presence of 4-DPS oxidizer (33). Disulfide-bonded ComGD-ComGF adducts were detected by immunoblotting on pilus purifications. The adducts were not detected in the presence of β-ME reducing agent.

Next, to strengthen these findings, we confirmed the validity of the structural model by experimentally probing the predicted ComGD-ComGF interface in the context of the pilus. To do this, we used an *in vivo* disulfide crosslinking approach (32). Guided by our pilus model, we chose two pairs of residues in ComGD (Leu_52_ and Gly_118_) and ComGF (Asp_47_ and Gln_96_), which fall within disulfide crosslinking distance. To facilitate the detection of minor pilins, we constructed *S. sanguinis* single and double mutants in a strain that constitutively expresses T4dP (17), with Cys substitutions in ComGD_L52C_/ComGF_D47C_ (Fig. 6A) and ComGD_G118C_/ComGF_Q96C_ (Fig. 6B). After shearing, filaments were briefly treated with thiol-specific oxidizer 4,4-dipyridyl disulfide (4-DPS) to promote the formation of disulfide bonds (33). Using anti-ComGD and anti-ComGF antibodies, we detected disulfide-bonded ComGD-ComGF adducts by immunoblotting in each of the two double mutants, but not in the respective single mutants (Fig. 6). Critically, under reducing electrophoresis conditions—in the presence of β-mercaptoethanol (β-ME)—the two proteins migrate as monomers (Fig. 6). In conclusion, by confirming predicted ComGD-ComGF interactions within the pilus, these results validate the model of the filament tip, especially in the area important for DNA binding.

Taken together, these results show that T4dP use an interface between two tip-located minor pilins—ComGD and ComGF—to promote DNA capture.

### Competent monoderms use a novel and conserved mode of DNA-binding involving the ComGD-ComGF interface

**Fig 7:**
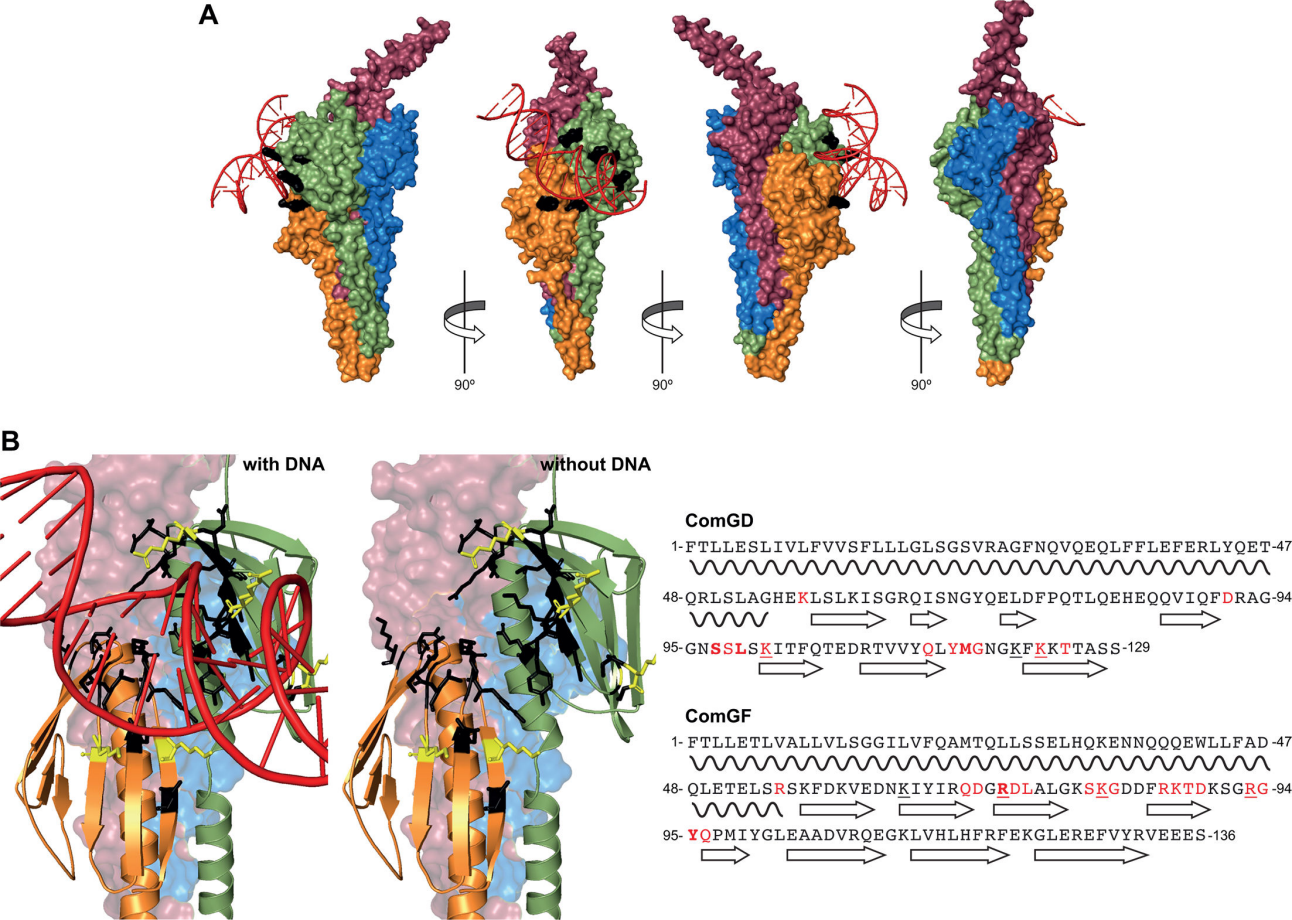
A structural model of the DNA/tip complex in *S. sanguinis* supports that the ComGD-ComGF interface binds DNA. (**A**) Structural model of the pilus tip in *S. sanguinis* interacting with DNA predicted by AlphaFold 3 (25). Since DNA uptake shows no sequence specificity in monoderms, we used for modeling a random 20 bp portion of pUC19 with 50% GC content. The surface representation clearly shows that DNA interacts only with the ComGD-ComGF interface on one side of the tip-located complex, involving the electropositive residues in these minor pilins that we identified as important for DNA (highlighted in black). (**B**) DNA-binding site as defined by analysis of the DNA/tip complex using PISA (34) and DNAproDB (35). Left panel, the residues involved in DNA-binding according to PISA and DNAproDB highlighted in black on a cartoon representation, or in yellow when they were also identified in our functional analysis. Right panel, summary of the residues involved in DNA-binding identified in the different analyses, displayed on the sequence of mature ComGD and ComGF. Bold, identified by DNAproDB. Red, identified by PISA. Underlined, identified in our functional analysis. Relevant structural features (α-helix or β-strand) are shown under the sequences.

Using the ability of AlphaFold 3 to predict the joint structure of complexes of proteins with a variety of ligands including nucleic acids (25), we predicted the structure of *S. sanguinis* T4dP interacting with DNA. Strikingly, as seen in Fig. 7A, the model (only the tip of the pilus is shown) corroborates and further strengthens the findings reported above. DNA clearly interacts only with one side of the tip-located complex of minor pilins, formed by the ComGD-ComGF interface (Fig. 7A). Furthermore, the electropositive residues in these two pilins identified as important for DNA capture—highlighted in black in Fig. 7A—are positioned at the interface with the DNA. Next, by analyzing the model using PISA (34) (Supplemental Data set 1) and DNAproDB (35) (Fig. S5), we precisely defined the DNA-binding site (Fig. 7B). Critically, it includes most residues identified in our phenotypic analysis—K_101_, K_123_ in ComGD, and R_73_, K_81_, R_93_ in ComGF—but also neighboring residues that are not electropositive (Fig. 7B). By providing a detailed view of how two minor pilins together bind DNA, this analysis highlights a novel mode of DNA-binding.

**Fig 8:**
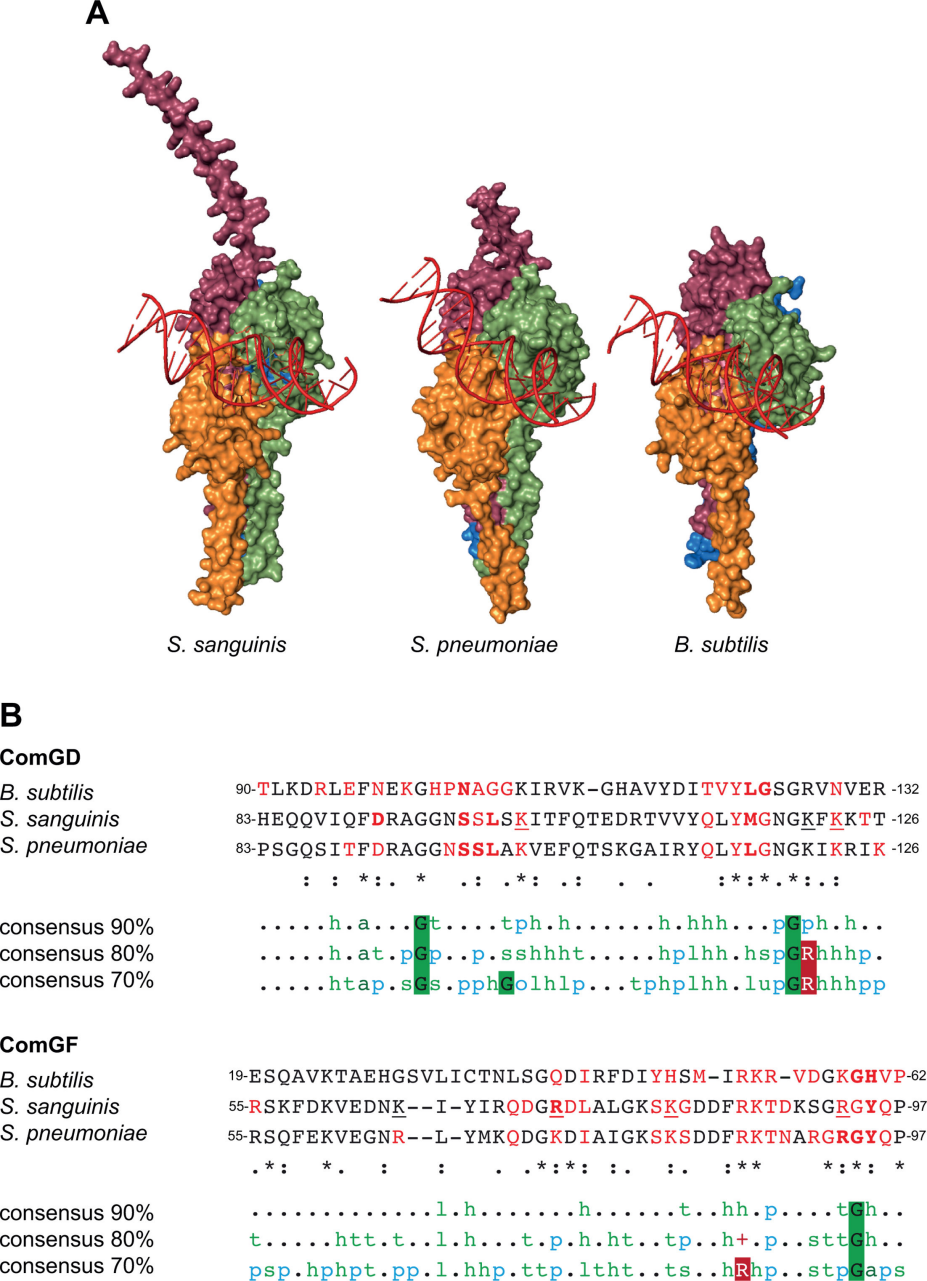
The novel mode of DNA binding we have identified in *S. sanguinis* is broadly conserved in monoderms expressing T4dP. (**A**) Structural models, predicted by AlphaFold 3 (25), of the Com pilus tips with bound DNA in *S. pneumoniae* and *B. subtilis* compared to *S. sanguinis*. The DNA always interacts exclusively with the ComGD-ComGF interface. (**B**) Comparison of the DNA-binding sites as defined by PISA (34) and DNAproDB (35) analyses of the DNA/tip complexes in *B. subtilis*, *S. pneumoniae,* and *S. sanguinis*. The residues involved in DNA-binding as defined by DNAproDB, PISA, and/or our functional analysis were identified in red, bold, and/or underlined, respectively. The sequences were aligned using Clustal Omega (36), with minor corrections for ComGF. The three bottom rows represent the 90%, 80%, and 70% consensus sequences—aligned to *B. subtilis*—formatted with MView (37) from MSA computed from the 1,794 ComGD and 2,580 ComGF entries in InterPro. h, hydrophobic (A, C, F, G, H, I, K, L, M, R, T, V, W, Y). t, turn-like (A, C, D, E, G, H, K, N, Q, R, S, T). a, aromatic (F, H, W, Y). p, polar (C, D, E, H, K, N, Q, R, S, T). s, small (A, C, D, G, N, P, S, T, V). o, alcohol (S, T). l, aliphatic (I, L, V). u, tiny (A, G, S). +, positive (H, K, R).

Finally, we determined whether this mode of DNA-binding is conserved in monoderms where T4dP are widespread (9). We generated models of tip-DNA complexes in other species where the Com pilus has been extensively studied, that is, *Bacillus subtilis* and *Streptococcus pneumoniae*. As shown in Fig. 8A, these models are strikingly similar to that from *S. sanguinis*, with DNA interacting specifically with the side of the pilus tip formed by ComGD-ComGF. Bioinformatic analyses of these models with PISA and DNAproDB show that the DNA-binding sites are the same as in *S. sanguinis*. Global multiple sequence alignments (MSAs) of the 1,794 ComGD and 2,580 ComGF proteins in InterPro using Clustal Omega (36) show that the residues at the ComGD-ComGF interface important for DNA-binding are broadly conserved (Fig. 8B).

Taken together, these results suggest that T4dP—found in hundreds of species of monoderms—use a conserved mode of DNA-binding, implicating two interacting minor pilins located at the pilus tip. This mode of DNA capture is strikingly different from that in diderms.

## DISCUSSION

Transformation is an important and widespread mechanism of HGT in bacteria, discovered almost 100 years ago (38), which allows competent species to acquire new genetic material (1). This multi-step process involves the capture of free DNA from the extracellular milieu by T4P. How T4P bind DNA remains poorly understood. In a few diderm species, which use T4aP for transformation, minor subunits that directly bind DNA have been identified (11, 12). In monoderms, which use a distinct T4P subtype (T4dP) (3), the molecular basis for DNA binding remains unknown. Here, we used *S. sanguinis*—a model monoderm for studying T4F (39)—to define how T4dP interact with DNA. This led to important findings on key aspects of T4F biology.

The main finding of this study is the unravelling of the mechanism of DNA-binding by T4dP, which appears conserved in monoderms. Like in competent diderms—where DNA-binding minor subunits of T4aP such as ComP and FimT have been identified (11, 12)—we show that minor pilins are involved in DNA capture by T4dP. This strongly suggests that in all the species using T4P for DNA capture—virtually all known competent bacteria except *Helicobacter*—specific pilin subunits will act as DNA receptors. However, the similarity ends there, and the modes of DNA-binding uncovered so far are unexpectedly different. First, in contrast to diderms where DNA-binding is mediated by single pilins (11, 12), monoderms use the interface between two different minor pilins. Both ComGD and ComGF contribute to DNA binding, and it is only by altering residues in both subunits that competence can be abolished. Although this remains purely speculative, it is likely that this mechanism has evolved by involving first one pilin in DNA-binding, which was then strengthened by co-opting the second one. Second, ComGD and ComGF are part of a tip-located complex of four pilins required for T4dP biogenesis, while ComP and FimT are dispensable for piliation and most likely randomly distributed along T4aP (11, 12). Indeed, in the latter filaments, the tip is already occupied by a large adhesin (PilC/PilY1) interacting with a complex of four broadly conserved minor pilins (24). This suggests that different T4P might have evolved the capacity to bind DNA independently during their evolutionary history. Third, although surface-exposed residues are always important, the different pilins playing a role in DNA capture appear to interact with DNA using very different binding sites. This makes it likely that other DNA-binding mechanisms remain to be discovered, notably in bacteria that use T4cP for DNA capture such as *Micrococcus luteus* (40) where no homologs of the above DNA-binding pilins are found.

The finding that the complex of four minor pilins at the T4dP tip plays a direct role in their main biological function is of general significance for T4F, because of the analogy with tip-located complexes of four minor pilins in other widespread T4F such as T4aP (24) and type 2 secretion systems (T2SS) (23). This set of four minor pilins—important in filament biogenesis and likely to have a common evolutionary origin—has thus been functionalized for very different properties. They (i) interact with and present the PilC/PilY1 adhesin in T4aP (24) that promotes adhesion to different surfaces (ii), interact with protein effectors in T2SS (41) to promote their secretion in the extracellular milieu, and (iii) bind free DNA to promote its capture by T4dP. Critically, DNA binding does not use the most salient structural feature in the T4dP tip complex, that is, the unstructured tail in ComGG, whose role thus remains mysterious. This functionalization is yet another evolutionary mechanism in bacteria—in addition to the use of modular pilins grafted with diverse functional modules (42, 43)—that contributes to the exceptional versatility of T4F.

In conclusion, by identifying the molecular basis of DNA capture by a T4P subtype found in hundreds of monoderm species and showing that a tip-located complex of four minor pilins found in multiple T4F is involved, this work has general implications for T4F. Moreover, by illustrating the power of experimental approaches based on accurate structural models generated by artificial intelligence programs, this study paves the way for future investigations that will further improve our understanding of these fascinating filaments.

## MATERIALS AND METHODS

### Structure modeling and bioinformatics

We used AlphaFold 3 (25) on the AlphaFold server for modeling protein 3D structures. We invariably chose models with the best pTM (predicted template modeling score) and/or ipTM (interface predicted template modeling score). The quality of the complex models was estimated using the Structure Assessment service at the Swiss Institute of Bioinformatics (29). We used PyMOL 3.1 (Schrödinger) for molecular visualization and for generating figures. The interfaces in protein/DNA complexes were analyzed using PISA (34) and DNAproDB (35). Signatures in proteins were identified by scanning against the InterPro database (31) using InterProScan (21). MSA was performed using Clustal Omega (36) and reformatted using MView (37). Sequence logos were generated from MSA using WebLogo 3 (44). Residues for Cys crosslinking experiments were selected using a combination of (i) sequence conservation, (ii) co-evolution assessed by EVcouplings (45), and (iii) the Disulfide by Design 2.0 tool for disulfide engineering in proteins (46).

### Protein expression and purification in *E. coli*

*E. coli* DH5α was used for cloning. *E. coli* BL21(DE3) was used for protein expression and purification. Strains were grown in liquid or solid lysogeny broth (Difco), containing, when required, 100 µg/mL ampicillin.

To construct expression plasmids (listed in Table S1), we used standard molecular biology techniques (47). We used pMALX(E) to generate MBP fusions with *S. sanguinis* ComGC and ComEA encoded by synthetic genes codon-optimized for expression in *E. coli* (GeneArt). The portions of *comGC_SS_* encoding ComGC_23–94_, which was previously characterized structurally (30), or *comEA_SS_* encoding ComEA_160–226_, were PCR amplified (primers are listed in Table S2). PCR products were cut with *Eco*RI and *Hin*dIII, purified using QIAquick PCR purification kit (Qiagen), and cloned in pMALX(E) cut by the same enzymes. The resulting plasmids were verified by sequencing.

For protein purification, 1 L cultures grown at 37°C until OD_600_ 0.6 was induced with 0.3 mM isopropyl 1-thio ß-d-galactopyranoside (Sigma) overnight (O/N) at 16°C. Bacteria were harvested by centrifugation at 12,000 × *g* and resuspended in lysis buffer (50 mM Tris-HCl pH 8, 100 mM NaCl) or (50 mM Tris-HCl pH 7.5, 200 mM NaCl, 1 mM EDTA) for MBP-ComEA and MBP-ComGC, respectively. The lysis buffer was supplemented with 20 µg/mL DNase I (Sigma) and EDTA-free protease inhibitor cocktail (Roche). Cells were lysed using a French Press. The recombinant proteins were affinity-purified using Poly-Prep chromatography columns (Bio-Rad) loaded with amylose resin (BioLabs) and eluted using lysis buffer containing 10 mM maltose. MBP-ComEA was treated for 2 h at 30°C with 30 µg/mL of DNase I in the presence of 20 mM MgCl_2_. To remove maltose and DNase, proteins were buffer-exchanged in lysis buffer using prepacked disposable PD-10 columns (Cytiva). Then, after a second affinity purification, the eluted proteins were buffer-exchanged in EMSA buffer (25 mM Tris-HCl pH 8, 2.5 mM MgCl_2_, and 50 mM NaCl) and concentrated using Amicon Ultra centrifugal filters with a 10 kDa cut-off (Millipore).

### Testing DNA-binding ability of purified proteins

The DNA-binding ability of purified proteins was tested by performing agarose EMSA essentially as described previously (11). Briefly, 120 ng pUC19 was incubated for 30 min at room temperature with increasing concentrations of MBP-ComGC or MBP-ComEA in 10 µL EMSA buffer. DNA was then separated by gel electrophoresis on 0.8% agarose (Fisher) in Tris acetate-EDTA buffer and visualized after ethidium bromide staining.

### Construction of *S. sanguinis* strains and growth conditions

All *S. sanguinis* strains used in this study (Table S1) are derivatives of the 2908 throat isolate (48). *S. sanguinis* strains were grown at 37°C using Todd Hewitt (TH) broth (Difco). Plates, TH broth with 1.5% agar, were incubated in anaerobic jars (Oxoid) under anaerobic conditions. Liquid cultures in THTH—TH containing 0.05% Tween 80 (Merck) to limit bacterial clumping, and 100 mM HEPES (Euromedex) to prevent acidification of the medium—were grown statically under aerobic conditions. Strains, constructed as previously described (17), were all verified by PCR and sequencing. Genomic DNA was prepared from liquid cultures using the XIT genomic DNA from Gram-Positive Bacteria kit (G-Biosciences). PCR was done using high-fidelity DNA polymerase (Agilent). The unmarked mutants were constructed by transforming *S. sanguinis* in the absence of selective pressure, with a splicing PCR product in which the regions upstream and downstream of the engineered mutations were amplified using F1/R1 and F2/R2 primers (Table S2). For the 5Q mutant construction, we combined the mutations in *comGD* and *comGF* by splicing, using as templates the genomic DNA of *comGD_K101Q/121Q/K123Q_* and *comGF_R73Q/R93Q_*. For the Cys crosslinking experiments, we constructed *S. sanguinis* single and double Cys mutants in ComGD and ComGF in a 2908 derivative that constitutively expresses Com pili (17). This was done to facilitate immunodetection of ComGD and ComGF.

### Transformation of *S. sanguinis*

Competence in *S. sanguinis* strains was quantified as described previously (17). Briefly, after O/N growth, bacteria were diluted in THTH. Induction was performed with 300 ng/mL synthetic competence-stimulating peptide (CSP), and we used 100 ng of a purified PCR product encompassing the *rpsL* gene from Str^R^ 2908, a mutant spontaneously resistant to streptomycin. After bath-sonication using a Bioruptor (Diagenode) at medium amplitude for 60 s to break bacterial chains, we performed serial dilutions that were spread on plates with and without streptomycin. Frequencies were determined as number of Str^R^ transformants/total CFU.

For non-selective transformation, after O/N growth, bacteria were diluted 10^−7^ in pre-warmed THTH and incubated at 37°C for 2 h. We then induced competence with CSP as above, took a 330 µL aliquot to which we added 500 ng of transforming DNA. After incubation for 2 h at 37°C, bacteria were bath-sonicated before plating. If colonies were non-clonal, as verified by sequencing, bath-sonication was repeated.

### Assaying piliation in *S. sanguinis*

Com pili were purified as previously described (17). Briefly, liquid cultures grown O/N were used to inoculate pre-warmed THTH at OD_600_ 0.01 and grown until the OD_600_ reached 0.04–0.08. Pilus production was induced with CSP for 30 min. Bacteria were pelleted by centrifugation for 15 min at 4,149 × *g* at 4°C. Pili were sheared by resuspending bacterial pellets in ice-cold pilus buffer by repeated pipetting up and down. Bacteria were then pelleted by two rounds of centrifugation at 4°C for 10 min at 9,220 × *g*. Finally, pili were pelleted by ultracentrifugation at 100,000 × *g* for 1 h at 4°C and resuspended in pilus buffer by pipetting up and down.

Pilus preparations were analyzed by immunodetection of the major pilin ComGC. Immunoblotting was done as described previously (17). Briefly, proteins were separated by SDS-PAGE in Tris-glycine buffer (Euromedex). Gels were transferred onto Amersham Hybond ECL nitrocellulose membrane (GE Healthcare) and analyzed by immunoblotting using anti-ComGC as primary antibody (at 1/2,500 dilution) and anti-rabbit HRP-conjugated (GE Healthcare) as secondary antibody (at 1/10,000 dilution). Detection was performed using Amersham ECL Prime Western Blotting Detection Reagent (GE Healthcare).

Purified pili in the 5Q mutant were visualized by TEM after negative staining as described elsewhere (17). We used a Tecnai 200 kV electron microscope (Thermo Fisher Scientific) and a Oneview 16 MP camera (Gatan) to acquire images.

### Cys crosslinking experiments

Pilus purification for the Cys crosslinking experiments in strains that constitutively express T4dP was done as follows. Liquid cultures grown O/N in THTH were used to inoculate pre-warmed THTH at 10^−8^ dilution and grown statically until OD_600_ reached 0.6. As above, bacteria were pelleted by centrifugation, and pili were sheared after re-suspending bacterial pellets in PBS (Sigma) by repeated pipetting up and down. Sheared filaments, recovered after two rounds of centrifugation as above, were incubated with 200 µM of 4-DPS (Sigma) for 30 min on ice. Finally, pili were pelleted by ultracentrifugation and resuspended in PBS. Samples were treated with and without β-ME (Aldrich) and analyzed by immunoblotting, using anti-ComGD or anti-ComGF as primary antibodies (at 1/1,000 dilution). Detection was performed using SuperSignal West Atto Ultimate Sensitivity Chemiluminescent Substrate (Thermo Scientific).

## SUPPLEMENTAL MATERIAL

Data set S1 mbio.00851-25-s0001.xlsx Interfacing residues between ComGD, ComGF, and DNA as defined by PISA.

Supplemental material mbio.00851-25-s0002.pdf Supplemental tables and figures.

ASM does not own the copyrights to Supplemental Material that may be linked to, or accessed through, an article. The authors have granted ASM a non-exclusive, world-wide license to publish the Supplemental Material files. Please contact the corresponding author directly for reuse.
