# Supplementary material for: Unraveling the molecular mechanisms of DNA capture by the Com pilus in naturally transformable monoderm bacteria
Source: mBio. 2025 May 23;16(6):e00851-25. doi: 10.1128/mbio.00851-25 (PMC12153270; doi:10.1128/mbio.00851-25)
Supplement: Supplemental material — Supplemental tables and figures. [file mbio.00851-25-s0002.pdf]

Unravelling the molecular mechanisms of DNA capture by the Com pilus in naturally transformable monoderm bacteria

Jérémy Mom, Odile Valette, Laetitia Pieulle#, Vladimir Pelicic#

Laboratoire de Chimie Bactérienne, UMR7283 Aix-Marseille Université/CNRS, Institut de Microbiologie de la Méditerranée, Marseille, France

#Address correspondence to pieulle@imm.cnrs.fr; vladimir.pelicic@inserm.fr

Table S1. Bacterial strains and plasmids used in this study.

**Name**	**Description**	**Source**
***S. sanguinis* strains**		
2908	throat isolate of *S. sanguinis*, WT strain	(1)
comGD$_{K57Q}$	unmarked mutant expressing ComGD$_{K57Q}$	this study
comGD$_{K61Q}$	unmarked mutant expressing ComGD$_{K61Q}$	this study
comGD$_{R65Q}$	unmarked mutant expressing ComGD$_{R65Q}$	this study
comGD$_{R92Q}$	unmarked mutant expressing ComGD$_{R92Q}$	this study
comGD$_{K101Q}$	unmarked mutant expressing ComGD$_{K101Q}$	this study
comGD$_{K121Q}$	unmarked mutant expressing ComGD$_{K121Q}$	this study
comGD$_{K123Q}$	unmarked mutant expressing ComGD$_{K123Q}$	this study
comGD$_{K124Q}$	unmarked mutant expressing ComGD$_{K124Q}$	this study
comGD$_{K121Q/K123Q}$	unmarked double mutant expressing ComGD$_{K121Q/K123Q}$	this study
comGD$_{K101Q/121Q/K123Q}$	unmarked triple mutant expressing ComGD$_{K101Q/K121Q/K123Q}$	this study
comGE$_{R63Q}$	unmarked mutant expressing ComGE$_{R63Q}$	this study
comGE$_{K66Q}$	unmarked mutant expressing ComGE$_{K66Q}$	this study
comGE$_{K69Q}$	unmarked mutant expressing ComGE$_{K69Q}$	this study
comGE$_{K85Q}$	unmarked mutant expressing ComGE$_{K85Q}$	this study
comGF$_{K57Q}$	unmarked mutant expressing ComGF$_{K57Q}$	this study
comGF$_{K65Q}$	unmarked mutant expressing ComGF$_{K565Q}$	this study
comGF$_{R69Q}$	unmarked mutant expressing ComGF$_{R69Q}$	this study
comGF$_{R73Q}$	unmarked mutant expressing ComGF$_{R73Q}$	this study
comGF$_{K81Q}$	unmarked mutant expressing ComGF$_{K81Q}$	this study
comGF$_{R93Q}$	unmarked mutant expressing ComGF$_{R93Q}$	this study
comGF$_{R108Q}$	unmarked mutant expressing ComGF$_{R108Q}$	this study
comGF$_{K112Q}$	unmarked mutant expressing ComGF$_{K112Q}$	this study
comGF$_{R119Q}$	unmarked mutant expressing ComGF$_{R119Q}$	this study
comGF$_{K122Q}$	unmarked mutant expressing ComGF$_{K122Q}$	this study
comGF$_{R73Q/R93Q}$	unmarked double mutant expressing ComGF$_{R73Q/R93Q}$	this study
5Q	unmarked quintuple mutant expressing ComGD$_{K101Q/K121Q/K123Q}$ ComGF$_{R73Q/R93Q}$	this study
comGG$_{\Delta 94\text{-}122}$	unmarked mutant expressing a tail-less ComGG$_{\Delta 94\text{-}122}$	this study

comGG$_{K52Q}$	unmarked mutant expressing ComG*G*$_{K52Q}$	this study
comGG$_{K64Q}$	unmarked mutant expressing ComG*G*$_{K64Q}$	this study
comGG$_{R68Q}$	unmarked mutant expressing ComG*G*$_{R68Q}$	this study
comGG$_{K72Q}$	unmarked mutant expressing ComGG$_{K72Q}$	this study
P$_{ldh}$ *comG* P$_{ldh}$ *comC*	2908 derivative expressing Com pili constitutively	(2)
comGD$_{L52C}$	unmarked mutant expressing ComGD$_{L52C}$	this study
comGF$_{D47C}$	unmarked mutant expressing ComGF$_{D47C}$	this study
comGD$_{L52C}$ *comGF*$_{D47C}$	unmarked double mutant expressing ComGD$_{L52C}$/ComGF$_{D47C}$	this study
comGD$_{G118C}$	unmarked mutant expressing ComGD$_{G118C}$	this study
comGF$_{Q96C}$	unmarked mutant expressing ComGF$_{Q96C}$	this study
comGD$_{G118C}$ *comGF*$_{Q96C}$	unmarked double mutant expressing ComGD$_{G118C}$/ComGF$_{Q96C}$	this study
5Q	unmarked quintuple mutant expressing ComGD$_{K101Q/K121Q/K123Q}$ ComGF$_{R73Q/R93Q}$	this study
Str^{R} 2908	spontaneous mutant resistant to streptomycin (*rpsL*$_{A167G}$)	(1)
***E. coli* strains**		
DH5α	used for cloning	
BL21(DE3)	used for protein expression/purification	
**plasmids**		
pMALX(E)	used for cytoplasmic expression of proteins fused to MBP	(3)
pMA-T-*comGC*$_{ss}$	synthetic *S. sanguinis comGC* in pMA-T, codon-optimized for expression in *E. coli*	(4)
pMALX-*comGC*$_{ss}$	pMALX(E) derivative for expression of MBP-ComGC$_{23-94}$	this study
pMA-RQ-*comEA*$_{ss}$	synthetic *S. sanguinis comEA* in pMA-RQ, codon-optimized for expression in *E. coli*	this study
pMALX-*comEA*$_{ss}$	pMALX(E) derivative for expression of MBP-ComEA$_{160-226}$	this study

P$_{ldh}$, constitutive promoter of the gene encoding the *S. sanguinis* 2908 lactate dehydrogenase.

MBP, maltose-binding protein.

Table S2. Primers used in this study.

**Name**	**Sequence***
**Construction of unmarked *S. sanguinis* mutants**	
ComGD-F1	AGGAGATCGGAGCAGACATG
ComGD-R2	AGTCCGACTTACCAGCTTCG
ComGD$_{K57Q}$-F2	GTTTAGCCGGTCATGAGCAACTGTCTCTCAAG
ComGD$_{K57Q}$-R1	CTTGAGAGACAGTTGCTCATGACCGGCTAAAC
ComGD$_{K61Q}$-F2	GGTCATGAGAAACTGTCTCTCCAGATTTCAGG
ComGD$_{K61Q}$-R1	CTGAAATCTGGAGAGACAGTTTCTCATGACC
ComGD$_{R65Q}$-F2	CTCAAGATTTCAGGCCAGCAGATTTCCAATG
ComGD$_{R65Q}$-R1	CATTGGAAATCTGCTGGCCTGAAATCTTGAG
ComGD$_{R92Q}$-F2	GTCATCCAGTTTGACCAGGCCGGTGGAAATTC
ComGD$_{R92Q}$-R1	GAATTTCCACCGGCCTGGTCAAACTGGATGAC
ComGD$_{K101Q}$-F2	CCTCACTCAGCCAAATTACTTTTCAGACGG
ComGD$_{K101Q}$-R1	CCGTCTGAAAAGTAATTTGGCTGAGTGAGG
ComGD$_{K121Q}$-F2	GGAATGGCCAGTTTAAAAAGACGACAGC
ComGD$_{K121Q}$-R1	GCTGTCGTCTTTTTAAACTGGCCATTCC
ComGD$_{K123Q}$-F2	GGAATGGCAAGTTTCAAAAGACGACAGCTT
ComGD$_{K123Q}$-R1	AAGCTGTCGTCTTTTGAAACTTGCCATTCC
ComGD$_{K124Q}$-F2	GGAATGGCAAGTTTAAACAGACGACAGCTTC
ComGD$_{K124Q}$-R1	GAAGCTGTCGTCTGTTTAAACTTGCCATTCC
ComGD$_{K121Q/K123Q}$-F2	GGAATGGCCAGTTTCAAAAGACGACAGCTTC
ComGD$_{K121Q/K123Q}$-R1	GAAGCTGTCGTCTTTTGAAACTGGCCATTCC
ComGE-F1	AGGAGATCGGAGCAGACATG
ComGE-R2	CAGAGCGTTTTGGATATTCTGG
ComGE$_{K63Q}$-F2	GGAATTACTGTACAACAGCTTAAGACAGATAAG
ComGE$_{K63Q}$-R1	CTTATCTGTCTTAAGCTGTTGTACAGTAATTCC
ComGE$_{K66Q}$-F2	GTACGTCAGCTTCAGACAGATAAGCAGCTG
ComGE$_{K66Q}$-R1	CAGCTGCTTATCTGTCTGAAGCTGACGTAC
ComGE$_{K69Q}$-F2	GCTTAAGACAGATCAGCAGCTGCTAGTCTATC
ComGE$_{K69Q}$-R1	GATAGACTAGCAGCTGCTGATCTGTCTTAAGC
ComGE$_{K85Q}$-F2	GTCATCCGTGTTCAACAACCTTAAAGTCAAG
ComGE$_{K85Q}$-R1	CTTGACTTTAAGGTTGTTGAACACGGATGAC
ComGF-F1	CAGCGGTGGTCAAGGTAGTA
ComGF-R2	AGTGTGCTCATCCGGACTAG
ComGF$_{K57Q}$-F2	GCTTTCGCGAAGTCAATTTGATAAGGTTG
ComGF$_{K57Q}$-R1	CAACCTTATCAAATTGACTTCGCGAAAGC
ComGF$_{K65Q}$-F2	GGTTGAAGACAATCAAATCTACATCAGAC
ComGF$_{K65Q}$-R1	GTCTGATGTAGATTTGATTGTCTTCAACC
ComGF$_{R69Q}$-F2	CAATAAAATCTACATCCAACAAGATGGCAGGG
ComGF$_{R69Q}$-R1	CCCTGCCATCTTGTTGGATGTAGATTTTATTG
ComGF$_{R73Q}$-F2	CAGACAAGATGGCCAGGACTTGGCCTTGGG
ComGF$_{R73Q}$-R1	CCCAAGGCCAAGTCCTGGCCATCTTGTCTG
ComGF$_{K81Q}$-F2	CTTGGGTAAATCCCAGGGAGATGATTTCCG
ComGF$_{K81Q}$-R1	CGGAAATCATCTCCCTGGGATTTACCCAAG
ComGF$_{R93Q}$-F2	GATAAGAGCGGTCAAGGCTACCAGCCCATG
ComGF$_{R93Q}$-R1	CATGGGCTGGTAGCCTTGACCGCTCTTATC
ComGF$_{R108Q}$-F2	GAAGCAGCTGACGTCCAACAGGAAGGTAAG
ComGF$_{R108Q}$-R1	CTTACCTTCCTGTTGGACGTCAGCTGCTTC
ComGF$_{K112Q}$-F2	CCGTCAGGAAGGTCAACTTGTCCATCTTC
ComGF$_{K112Q}$-R1	GAAGATGGACAAGTTGACCTTCCTGACGG
ComGF$_{R119Q}$-F2	CCATCTTCATTTTCAGTTTGAAAAAGGTTTAG
ComGF$_{R119Q}$-R1	CTAAACCTTTTTCAAACTGAAAATGAAGATGG
ComGF$_{K122Q}$-F2	CGCTTTGAACAAGGTTTAGAGAGGGAGTTCG
ComGF$_{K122Q}$-R1	CGAACTCCCTCTCTAAACCTTGTTCAAAGCG
5Q-F1	AGGAGATCGGAGCAGACATG
5Q-R1	GCTGACGTACAGTAATTCCG

5Q-F2	TCTGCTGGAAGCTCTGGTGG
5Q-R2	AGTGTGCTCATCCGGACTAG
ComGG-F1	GGGCTGGCTTCAATCAGGTTC
ComGG-R2	CCAGTTTTGAAAACTTTGGCGG
ComGG$_{K52Q}$-F2	CAAAAGCTACAGCCCAGGATGATAGCGGTG
ComGG$_{K52Q}$-R1	CACCGCTATCATCCTGGGCTGTAGCTTTTG
ComGG$_{K64Q}$-F2	GTTTGAGCAGGGCCAGGCTGTCTACCGCAGG
ComGG$_{K64Q}$-R1	CCTGCGGTAGACAGCCTGGCCCTGCTCAAAC
ComGG$_{R68Q}$-F2	CAAGGCTGTCTACCAAAGGCAAGGGAAGAATC
ComGG$_{R68Q}$-R1	GATTCTTCCCTTGCCTTTGGTAGACAGCCTTG
ComGG$_{R72Q}$-F2	CTACCGCAGGCAAGGGCAGAATCTTGAAATC
ComGG$_{R72Q}$-R1	GATTTCAAGATTCTGCCCTTGCCTGCGGTAG
ComGG$_{\Delta 94-122}$-F2	CCTTTACTTTTAGTACTTAAAAGAAAGATGAGGGTAAAGC
ComGG$_{\Delta 94-122}$-R1	GCTTTACCCTCATCTTTCTTTTAAGTACTAAAAGTAAAGG
ComGD$_{L52C}$-F2	GACGCAGAGGCTGAGTTGTGCCGGTCATGAG
ComGD$_{L52C}$-R1	CTCATGACCGGCACAACTCAGCCTCTGCGTC
ComGD$_{G118C}$-F2	CAGCTTTATATGTGTAATGGCAAGTTTAAAAAG
ComGD$_{G118C}$-R1	CTTTTTAAACTTGCCATTACACATATAAAGCTG
ComGF$_{D47C}$-F2	GCTTTTGTTTGCCTGTCAACTGGAGACGGAG
ComGF$_{D47C}$-R1	CTCCGTCTCCAGTTGACAGGCAAACAAAAGC
ComGF$_{Q96C}$-F2	CGGTCGCGGCTACTGTCCCATGATTTATGG
ComGF$_{Q96C}$-R1	CCATAAATCATGGGACAGTAGCCGCGACCG

Generating a PCR product for quantifying competence

rpsL-F	GGCAGGTGTAGCTGTCTTTG
rpsL-R	CTCTTGCTCCATCCAGTCCA

Construction of plasmids for protein expression/purification

comGC$_{SS}$-F	gcgc**gaattc**AATCTGACCAAACAGAAAGATGCAGTTAGC
comGC$_{SS}$-R	ggg**aagctt**TTAATTTGCAACGGTCTGGGTTTCACCG
comEA$_{SS}$-F	gctagc**gaattc**GGTAAAAGCGATAAACTGAATC
comEA$_{SS}$-R	agtgcc**aagctt**TTAATCCACGGTCACATATTCTTTCAG

*Regions of complementarity for splicing PCR are underlined. Overhangs are in lower case. Restriction sites are in bold.

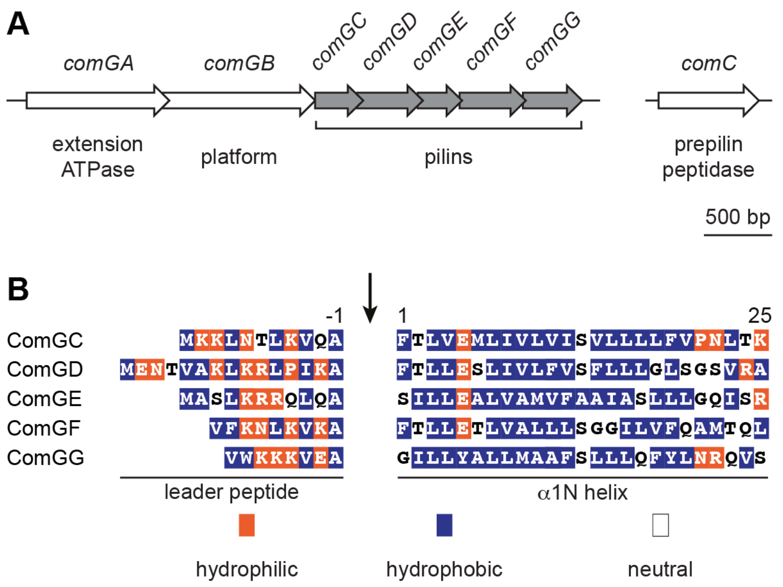

Fig. S1. The Com pilus machinery in *S. sanguinis* 2908. A) Genomic organization of the genes involved in the synthesis of the Com pilus. The genes are drawn to scale. **B**) Alignment of the N-terminal class 3 signal peptides in the five Com pilins. The leader peptides – cleaved by the PPase ComC after a conserved Ala (indicated by the vertical arrow) (2) – contain a majority of hydrophilic (shaded in orange) and neutral (no shading) residues. The mature proteins start with a tract of predominantly hydrophobic residues (shaded in blue), which correspond to the protruding N-terminal half of an extended α-helix (α1N) that is the main assembly interface of pilins within T4F.

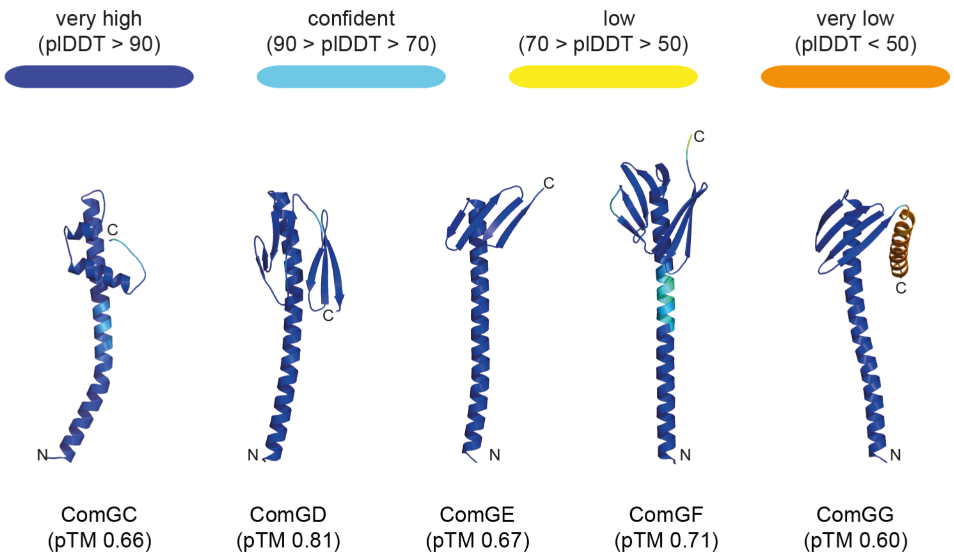

Fig. S2. AlphaFold 3 structural models of the five pilins highlight typical "lollipop" architectures. We modelled the mature pilins in which the SP3 cleaved by ComC (see Fig. S1) was manually removed. The predictions are highly accurate as shown by coloring by pLDDT – per-atom confidence score – as in the AlphaFold Protein Structure Database (AFDB) (5). The predicted template modelling scores (pTM) are indicated within parentheses. The last 30 residues of ComGG are the only portion with very low accuracy.

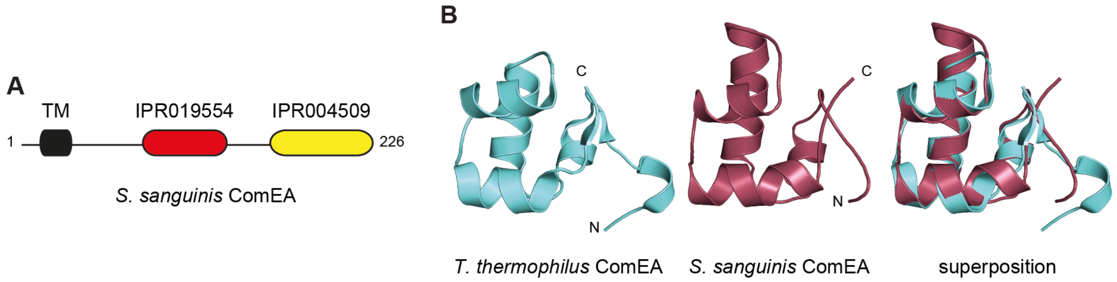

Fig. S3. The ComEA DNA receptor in *S. sanguinis* involved in the late stages of DNA uptake. **A**) In *S. sanguinis*, ComEA is a membrane-anchored bimodular protein where only the C-terminal module (IPR004509) corresponds to ComEA. TM, trans-membrane domain. IPR019554, Soluble ligand-binding β-grasp domain. **B**) The C-terminal module of *S. sanguinis* ComEA, modelled using AlphaFold 3, is structurally similar (RMSD 0.605 Å) to the ComEA crystal structure of *T. thermophilus* (PDB entry 2DUY).

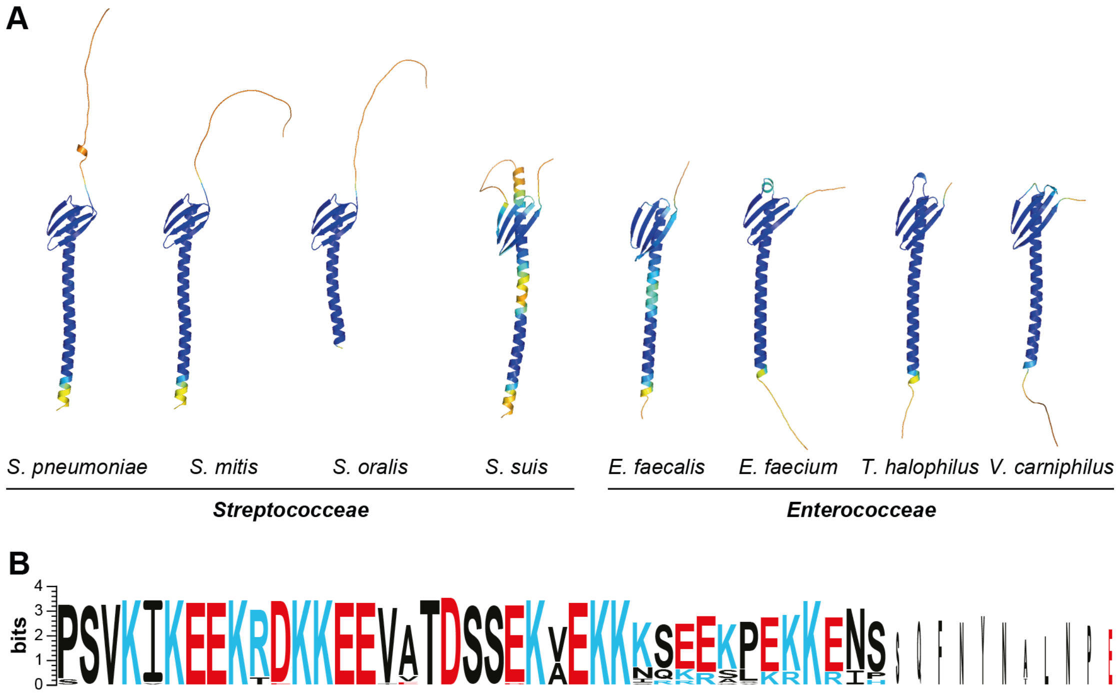

Fig. S4. Charged C-terminal tails are a conserved feature in *Streptococcus*-type ComGG (IPR047665). A) Structural models from the AFDB (5) for IPR047665 entries in taxa covering most of the diversity in which *Streptococcus*-type ComGG are found. *Streptococcus pneumoniae* (A0A064C498), *Streptococcus mitis* (A0A081PSG0), *Streptococcus oralis* (A0A081R7G1), *Streptococcus suis* (A0A0M9FP66), *Enterococcus faecalis* (A0A2R6UDT3), *Enterococcus faecium* (A0A133CQY1), *Tetragenococcus halophilus* (A0A3G5FGM1), *Vagococcus carniphilus* (A0A430B3T3). All these pilin – colored by pLDDT (see Fig. S2) – display C-terminal tails with very low prediction accuracy. **B**) The ComGG tails are usually highly charged. Sequence logo generated from a multiple sequence alignment of 39 IPR047665 entries for *S. pneumoniae*. Charged residues are colored in blue (electropositive) or red (electronegative).

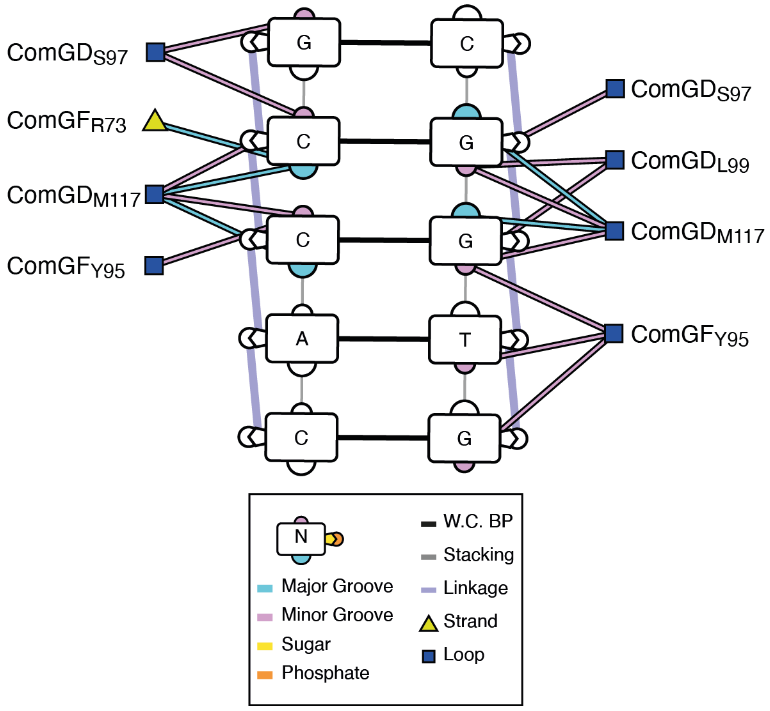

Fig. S5. Bioinformatic analysis of the structural model of the DNA/tip complex in *S. sanguinis* using DNAproDB. The residue contact map – generated using DNAproDB (6) – shows individual nucleotide-residue interactions. The DNA is displayed as a graph, where nucleotides represent nodes, and edges between them indicate backbone links, base pairing, or base stacking. Protein residues are displayed as small nodes with the node shape and color representing residue secondary structure. Edges between protein residue and nucleotide nodes represent interactions between the two, and which DNA moiety is involved.

References

1. Gurung I, Spielman I, Davies MR, Lala R, Gaustad P, Biais N, Pelicic V. 2016. Functional analysis of an unusual type IV pilus in the Gram-positive *Streptococcus sanguinis*. Mol Microbiol 99:380-392.
2. Mom J, Chouikha I, Valette O, Pieulle L, Pelicic V. 2024. Systematic functional analysis of the Com pilus in *Streptococcus sanguinis*: a minimalistic type 4 filament dedicated to DNA uptake in monoderm bacteria. mBio 15:e02667-23.
3. Moon AF, Mueller GA, Zhong X, Pedersen LC. 2010. A synergistic approach to protein crystallization: combination of a fixed-arm carrier with surface entropy reduction. Protein Sci 19:901-913.
4. Sheppard D, Berry JL, Denise R, Rocha EPC, Matthews SJ, Pelicic V. 2020. The major subunit of widespread competence pili exhibits a novel and conserved type IV pilin fold. J Biol Chem 295:6594-6604.
5. Varadi M, Anyango S, Deshpande M, Nair S, Natassia C, Yordanova G, Yuan D, Stroe O, Wood G, Laydon A, et al. 2022. AlphaFold Protein Structure Database: massively expanding the structural coverage of protein-sequence space with high-accuracy models. Nucleic Acids Res 50:D439-D444.
6. Mitra R, Cohen AS, Sagendorf JM, Berman HM, Rohs R. 2025. DNAproDB: an updated database for the automated and interactive analysis of protein-DNA complexes. Nucleic Acids Res 53:D396-D402.
